# Data-Driven Methods for the Estimation of Leaf Water and Dry Matter Content: Performances, Potential and Limitations

**DOI:** 10.3390/s20185394

**Published:** 2020-09-21

**Authors:** Bin Yang, Hui Lin, Yuhao He

**Affiliations:** 1 College of Electrical and Information Engineering, Hunan University, Changsha 410082, China; binyang@hnu.edu.cn (B.Y.); linhui1965@126.com (H.L.); 2 Key Laboratory of Visual Perception and Artificial Intelligence of Hunan Province, Hunan University, Changsha 410082, China

**Keywords:** leaf equivalent water thickness, leaf mass per area, data-driven method, machine learning, vegetation index, LOPEX and ANGERS

## Abstract

Leaf equivalent water thickness (EWT) and dry matter content (expressed as leaf mass per area (LMA)) are two critical traits for vegetation function monitoring, crop yield estimation, and precise agriculture management. Data-driven methods are widely used for remote sensing of leaf EWT and LMA because of their simplicity, satisfactory accuracy, and computation efficiency, such as the vegetation indices (VI)-based and machine learning (ML)-based methods. However, most of the data-driven methods are utilized at the canopy level, comparison of the performances of the data-driven methods at the leaf level has not been well documented. Moreover, the ML-based data-driven methods generally adopt leaf optical properties directly as their inputs, which may subsequently decrease their ability in remote sensing of leaf biochemical constituents. Performances of the ML-based methods cooperating with VI are rarely evaluated. Using the independent LOPEX and ANGERS datasets, we compared the performances of three data-driven methods: VI-based, ML-reflectance-based, and ML-VI-based methods, for the estimation of leaf EWT and LMA. Three sampling strategies were also utilized for evaluation of the generalization of these data-driven methods. Our results evidenced that ML-VI-based methods were the most accurate among these data-driven methods. Compared to the ML-reflectance-based and VI-based methods, the ML-VI-based model with support vector regression overall reduced errors by 5.7% (41.5%) and 1.8% (12.4%) for the estimation of leaf EWT (LMA), respectively. The ML-VI-based model inherits advantages of vegetation indices and ML techniques, which made it sensitive to changes of leaf biochemical constituents and capable of solving nonlinear tasks. It is thus recommended for the estimation of EWT and LMA at the leaf level. Moreover, its performance can further be enhanced by improving its generalization ability, such as adopting techniques on the selection of better wavelengths and definition of new vegetation indices. These results thus provided a prior knowledge of the data-driven methods and can be helpful for future studies on the remote sensing of leaf biochemical constituents.

## 1. Introduction

Leaf water and dry matter content are among the most important biochemical indicators that determine plant photosynthetic capacity and ecosystem processes [1,2,3]. Quantifying changes in these leaf biochemical indicators is critical for plant function monitoring, crop yield estimation, and precise agriculture management. By definition, leaf water content is parameterized as equivalent water thickness (EWT), whereas leaf dry matter content is specified as leaf mass per area (LMA) [4]. EWT and LMA can be calculated as the amount of water and dry mass per leaf area, respectively. They could be measured by both laboratory destructive measurements and remote sensing techniques. Being fast, effective, and nondestructive, remote sensing techniques using leaf optical properties have become a popular approach for the estimation of EWT and LMA [5,6,7,8].

With the development of hyperspectral instrumentation and remote sensing theory, quite a few approaches have been proposed for the estimation of EWT and LMA. These approaches can be broadly categorized as two types: physical-based and data-driven methods [9]. Physical-based methods minimize the difference between the measured and modeled leaf optical properties using radiative transfer models. There are several radiative transfer models that could be used for this purpose, such as the PROSPECT [10,11], LIBERTY [12,13], and SLOPE [14] models. Among them, PROSPECT is the most widely used because of its relatively simple and satisfactory performances. Several inversion algorithms have also been proposed, including look-up-table [15] and iterative optimizations methods [4,16]. These algorithms were proposed based on the assumption that the leaf specific absorption spectra are fixed for all vegetation species, and cannot account for the spectral variability of leaf biochemical constituents [17]. Moreover, they may suffer from ill-posed problems and high computation cost.

Data-driven methods are built based on the statistical relationship between leaf biochemical constituents and leaf optical properties. The statistical relationship is generally calibrated using a training dataset with regression techniques, ranging from simple linear regression [18], partial least square regression (PLSR) [19], to complex machine learning (ML) techniques, such as the support vector machine regression [2], artificial neural networks [20], and random forest regression [21]. Leaf optical properties include, but are not limited to, leaf reflectance, its derivates and combinations, such as the vegetation indices (VI) [22,23], and red-edge positions [24]. Data-driven methods are generally simple to use, accurate, and computationally effective, and thus are preferred for fast estimation of leaf biochemical constituents in agricultural applications.

Vegetation indices for the estimation of EWT and LMA are combinations of reflectances at two or more wavebands in the 900–2400 nm spectral region [18], where strong/weak absorption of water and/or dry matter occurred. Therefore, wells and shoulders of water absorption around 970, 1200, 1500, and 2200 nm are densely utilized for the estimation of EWT [25]. As for LMA, its accurate estimation is challenging because of the predominant water absorption [6,26]. Studies usually adopt the absorption of the C-H bond stretch at around 1700 nm to suppress the influence of water [27]. Such characteristics explain why the EWT-related indices usually involve reflectance at around 970, 1200, 1500, or 2200 nm [28,29,30,31,32], whereas the LMA-related indices usually involve reflectance at around 1700 nm [22,23,27]. These indices have various types, varying from simple ratio, normalized difference to complex mathematical combinations. Generally, the types of simple ratio and normalized differences are easy to understand and use. Therefore, they are widely used for the estimation of EWT and LMA.

In the framework of data-driven methods, a linear or an exponential function built between VI and leaf biochemical constituents is among the simplest means (i.e., VI-based method), whereas a nonlinear relationship built using ML techniques are among the most complicated means. ML techniques have the advantage of solving nonlinear tasks, and have been widely used in remote sensing of leaf biochemical constituents in recent years. Notably, most of the data-driven methods focused on the canopy level [33,34,35], and only a few studies focused on the leaf level [2,7]. Accurate estimation of leaf biochemical constituents using leaf optical properties is fundamental for understanding what insides the leaf. According to the best of our knowledge, evaluation of these data-driven methods for the estimation of EWT and LMA at the leaf level has never been well documented.

Moreover, most of the ML techniques were adopted by using leaf optical properties at a wide spectral region as inputs (i.e., ML-reflectance-based method). For instance, Ref. [7] built a neural network, and Ref. [2] utilized a support vector regression (SVR) implementation for the estimation of EWT and LMA using leaf reflectance and transmittance at wavelengths covering the 900–2400 nm spectral region. Using leaf optical properties at such a wide spectral region may not always be helpful. One study reported that including leaf optical properties in the 900–1300 nm spectral region could decrease the accuracy of LMA estimation [2]. Leaf optical properties in a spectral region are highly correlated [18], indicating that the leaf reflectance and/or transmittance at a specific wavelength may be enough for the representation of that at a wide spectral region. Combinations of leaf optical properties at two or more wavelengths can enhance the sensitivity to leaf biochemical constituents, as the VI-based method does. Another study suggested that incorporating ML techniques with VI can help to improve the performance of remote sensing of leaf biochemical constituents. Ref. [21] adopted 45 established VI as inputs to the random forest (RF) for the estimation of leaf chlorophyll content, and found that the error could be significantly reduced compared to the standard regression. However, incorporating ML techniques with VI for the estimation of EWT and LMA has rarely been reported.

Therefore, this paper focuses on the remote sensing of EWT and LMA at the leaf level using data-driven methods. The objectives of this paper are to: (1) intercompare the performances of the most widely used data-driven methods, i.e., the VI-based method and the ML-reflectance-based method, and the new method that incorporating ML techniques with VI (ML-VI-based method); (2) explore the potential and limitations of the data-driven methods. The most widely used VIs that are sensitive to leaf EWT and LMA, and the most popular ML techniques are used in this paper. The LOPEX and ANGERS datasets were adopted for evaluations of these data-driven methods at the leaf level. This study provided a prior knowledge of the data-driven methods and can be applied to future studies on the remote sensing of leaf biochemical constituents.

This paper is organized as follows. General description of the LOPEX and ANGERS datasets are given in Section 2.1. The most widely used VI and ML are introduced in Section 2.2 and Section 2.3. To achieve the objectives of this paper, three experiments are designed, as described in Section 2.4. Section 3 presents the results obtained with these experiments. Section 4 discusses the performance, potential, and limitations of the data-driven methods. Finally, the concluding remarks are provided in Section 5.

## 2. Materials and Methods

### 2.1. Description of the Experimental Datasets

In this study, two independent datasets were adopted, i.e., the LOPEX and ANGERS datasets. These two datasets were collected with synchronous measurements on leaf optics and leaf biochemical constituents. They represent the most popular and easy to access tool for remote sensing of leaf biochemistry, and have been widely used across the world. The LOPEX dataset was collected at the Joint Research Center of Italy (Ispra, Italy) in 1993 over 320 fresh leaf samples from 45 different species [36]. The ANGERS dataset was collected at INRA (National Institute of Agronomy) in Angers, France in 2003 over 276 leaf samples from 43 different species [37]. In the LOPEX and ANGERS experiments, fresh weights of leaf discs were measured before drying them in an oven at 85 °C for 48 h. After drying, they were reweighted to determine the corresponding EWT and LMA [37]. They recorded data over 596 leaves of multiple herbaceous woody species under a variety of spectra, vegetation structures, and biological components. The datasets are publicly available via http://opticleaf.ipgp.fr/index.php?page=database. A detailed description of the two datasets was documented in [36,37].

**Figure 1:**
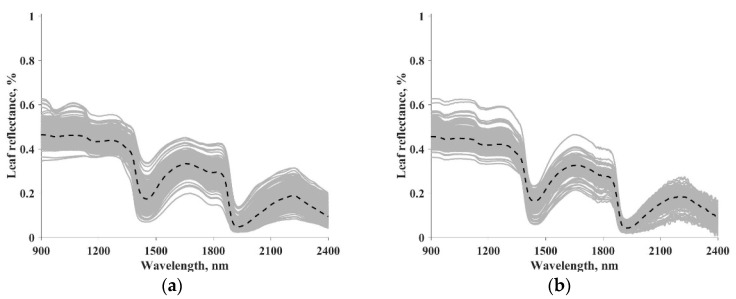
Spectrum of leaf reflectance from the LOPEX (**a**) and ANGERS (**b**) datasets. The dashed dark line represents the median spectrum.

In both datasets, leaf reflectance and transmittance in the 400–2500 nm spectral range with 1 nm step were measured in the laboratory spectrophotometers or field spectroradiometers equipped with integrating spheres [2]. In this study, we focused on EWT and LMA inversion and, therefore, the 900–2400 nm spectral range was selected for a higher sensitivity to the changes of EWT and LMA [2,16]. Leaf reflectance was used for calculation of vegetation indices and as input for data-driven models. Table 1 summarizes the statistical information of LOPEX and ANGERS datasets, including the number of samples and species, minimum, maximum, mean, and standard deviation of the EWT and LMA. Figure 1 illustrates the spectrum of leaf reflectance from the LOPEX and ANGERS datasets. Each gray line represents the reflectance of a specific leaf, whereas the dashed black line is the median spectrum.

### 2.2. Vegetation Indices

Ten vegetation indices, which have been reported to be most sensitive to leaf- and canopy-level EWT and LMA, were selected in this study. Table 2 shows these vegetation indices. Generally, these indices follow the type of simple ratio or normalized difference. They were calculated using leaf reflectances at two wavebands in the 900–2400 nm spectral region, where strong/weak absorption of water and/or dry matter occurred.

#### 2.2.1. Vegetation Indices Sensitive to EWT

Based on Colombo et al. [25], six vegetation indices proposed for EWT estimation were selected in this study. These EWT-related indices are water index (WI) [28], normalized difference water index (NDWI) [29], simple ratio water index (SRWI) [30], normalized difference infrared index (NDII) [38], moisture stress index (MSI) [32] and difference water index (DWI) [25]. The WI, SRWI, and MSI follow the type of simple ratio, whereas the rest follow the type of normalized difference. They were calculated by exploiting wells and shoulders of water absorption around 970, 1200, 1500, and 2200 nm, as shown in Table 2. All these indices have been widely used in the estimation of EWT at both leaf and canopy levels, as documented in [18,25,31].

#### 2.2.2. Vegetation Indices Sensitive to LMA

Four vegetation indices that are related to LMA estimation were selected in this study. These LMA-related indices are normalized difference for LMA (NDLMA) [23], normalized dry matter index (NDMI) [27], normalized difference (ND) [23], and ratio index (RI) [22]. Estimation of LMA has been reported to be challenging because of the predominant absorption of water [2,6]. These indices usually adopt a leaf reflectance around 1700 nm because of the absorption of C-H bond stretch, and another reflectance at other wavebands to suppress the influence of water. These indices have been reported to provide satisfactory performance when utilized for LMA estimation [22,23].

### 2.3. Machine Learning Techniques

#### 2.3.1. K-Nearest Neighbor (KNN)

KNN is a lazy supervised learning regression technique. It is fast and effective for high-dimensional data regression problems [39,40]. The basic idea behind KNN is finding a set of K samples that are most closed to the unknown sample based on a similarity measurement (e.g., Euclidean distance as used in this study) and predicting the value of the unknown sample using the average of the response variables of the K-nearest neighbors [41,42,43]. The parameter, K, has a significant impact on the performance of the KNN technique. A small K indicates that only a small portion of the training data that is most close to the unknown sample gives the prediction. This type of prediction could be impacted by noise or uncertainty within the training data. A large K can suppress the noise or uncertainty, but may introduce many unrelated learning samples.

#### 2.3.2. Partial Least Squares Regression (PLSR)

PLSR is an extension of multiple linear statistical techniques. It integrates the advantages of principal component analysis, canonical correlation analysis, and linear regression analysis [22,44,45]. It can effectively address the problem of providing good predictions in multivariate regression, even with a few training data and multiple-correlated input variables. The basic idea behind PLSR is reducing a large number of reflectances or their derivates to a few principal components (PCs), and making regression using several selected PCs [19,46].

The key of PLSR is to build a linear model as follows,

(1) y=x · β + ε

Here, *y* is the mean-centered vector of dependent variables (EWT and LMA). *x* represents the mean-centered vector of independent variables (reflectance). β and ε are regression coefficient and residual, respectively. In PLSR, the above principles are adopted on PCs of *x*.

The number of selected PCs has a great influence on the performance of the PLSR technique. A small number of selected PCs may cause data loss and thus underfitting occurs. These problems can be addressed by increasing the number of selected PCs, but may consequently cause overfitting and higher computation cost. In this paper, the PLSR2 model with the NIPALS algorithm was used.

#### 2.3.3. Support Vector Regression (SVR)

SVR is an important branch of support vector machines (SVMs) [20]. It can provide good regression performance because it transfers a low-dimensional nonlinear input to a high-dimensional linear output. The basic idea behind the SVR is finding a hyperplane that can fit all data (that is, all sample points have the smallest total deviation from the hyperplane) [47,48,49].

The key of SVR is to solve the following equation,

(2) $$\begin{array}{c} min\\ \omega ,b,\xi_{i},{\hat{\xi }}_{i} \end{array}\left(\frac{1}{2}{\left\|\omega \right\|}^{2}+C\overset{m}{\underset{i=1}{\sum }}\left(\xi_{i}+{\hat{\xi }}_{i}\right)\right)$$

$$s.t.\;y_{i}-\omega \Phi (x_{i})-b\leq \varepsilon +\xi_{i},$$

$$\omega \Phi (x_{i})+b-y_{i}\leq \varepsilon +{\hat{\xi }}_{i},$$

$$\xi_{i}\geq 0,{\hat{\xi }}_{i}\geq 0,i=1,2,\ldots ,m.$$

Here, ω is the normal vector of the linear function, b is the intercept. Φ represents a nonlinear transformation from the current dimension to a high-dimensional space, which could be specified by a kernel function. Slack variable $\xi_{i}$ and ${\hat{\xi }}_{i}$ correspond to the upper and lower parameters in which (ωΦ(x)+b) is allowed to deviate by an error, ε, and a cost, C. Finally, *x* is leaf reflectance and *y* is leaf EWT or LMA in this study.

A kernel function determines the distribution of sample points in the high-dimensional space, and is thus important for the SVR. In this study, the radial basis function (RBF) function was selected as the kernel function, which implies two critical parameters need to be optimized, C and γ. C is the cost parameter that is related to tolerance for error, whereas γ is a parameter unique to the RBF kernel function and affects the speed of model prediction. A large C implies that a large error cannot be tolerated, which consequently may result in overfitting. As for a small C, a large error is acceptable and underfitting may occur. The number of support vectors is adjusted to affect the speed of training and prediction by determining γ. A large γ indicates fewer support vectors, whereas a small γ indicates more support vectors.

#### 2.3.4. Random Forest (RF)

RF is a nonparametric ensemble machine learning algorithm based on multiple decision trees to train samples and achieve estimation. It is popular in the field of remote sensing due to its high accuracy and stability [50]. The basic idea behind the RF regression is that each decision tree is calculated separately on the dataset, the results are transmitted and the average thereof selected as the final prediction result [21].

The key to RF regression is to split regression trees. This process is done by choosing the input variable with the minimum Gini index, i.e.,

(3) $$I_{G}\left(t_{x_{i}}\right)=1-\overset{m}{\underset{i=1}{\sum }}f{\left(t_{x_{i}},j\right)}^{2}$$

Here, $f\left(t_{x_{i}},j\right)$ represents the proportion of observations with value $x_{i}$ belonging to leaf *j* as node *t*. $I_{G}$ is the corresponding Gini index.

Three parameters are required to be optimized at the RF regression process, number of decision trees, maximum depth, and terminal nodes. The number of decision trees needs to be maximum for a dense forest. The maximum depth of the decision tree is limited to avoid overfitting. The terminal nodes determine when the tree growth should be stopped. A large number of terminal nodes imply that tree growth is stopped after a few splits, which would result in underfitting, whereas a small number of terminal nodes could cause overfitting.

#### 2.3.5. Cross-Validation

In order to optimize the best set of ML parameters, all the ML techniques were validated using the K-fold cross-validation procedure. The K-fold cross-validation procedure randomly and equally divided the training data into five subsamples, among which four subsamples were used to calibrate the ML models while the other subsample was used as “out of bag” to calculate the prediction error. This process was repeated five times until each subsample has been used and only used once for calculation of the prediction error. In this study, the prediction error was parameterized as the RMSE. The best set of parameters for these ML techniques were selected when they provided the smallest RMSE. A detailed description of K-fold cross-validation is documented in [51]. For the KNN, the K was optimized within [1, 3, 5, 7, 10]. For the PLSR, the number of PCs was optimized within [2, 3, 4]. For the SVR, the C and γ were optimized within [10^−2^, 10^−1^, 1, 10, 100] and [10^−4^, 10^−3^, 10^−2^, 10^−1^, 1, 10], respectively. For the RF, number of decision trees, maximum depth, and terminal nodes were optimized within [10, 20, 30, 40, 50, 60, 70], [5, 7, 9, 11, 13, 15], and [1, 2, 3, 4, 5], respectively. All parameters of these ML techniques were optimized by the grid search function using the Python 3.6 implementation of the Scikit-Learn package.

### 2.4. Design of Experiments

In this study, three data-driven methods were applied to the LOPEX and ANGERS datasets, and their performances were compared to each other. These methods aimed at building an empirical relationship between leaf optical properties (i.e., leaf reflectance and its combinations) and leaf biochemical constituents (i.e., EWT and LMA). These three data-driven methods are:

**Method 1 (M1):**  *VI-based method, which builds a relationship between VI and EWT or LMA using a linear or an exponential function.*

**Method 2 (M2):**  *ML-reflectance-based method, which builds a relationship between leaf reflectance and EWT or LMA using ML techniques.*

**Method 3 (M3):**  *ML-VI-based method, which builds a relationship between VI and EWT or LMA using ML techniques.*

It is notable that linear and exponential functions were selected in M1 because of their wide applications in remote sensing of biochemical constituents, as documented in [18,33]. For each pair of leaf biochemical constituent and vegetation index, the regressed model with the highest accuracy was selected as the optimal model.

The performances of the data-driven methods depend on the similarity between the training and validation datasets. Generally, the performances are calculated after splitting the experimental datasets into two subsets, one for training and the other for validation, and the regressed models are not validated on a completely independent dataset. This may raise the question that if the regressed models can be applied to another independent dataset, which satisfactory performances are also provided. To answer this question, three sampling strategies are designed:

**Sampling strategy 1 (S1):**  *using the LOPEX dataset as the training dataset, instead of using the ANGERS dataset as the validation dataset.*

**Sampling strategy 2 (S2):**  *using the ANGERS dataset as the training dataset, instead of using the LOPEX dataset as the validation dataset.*

**Sampling strategy 3 (S3):**  *mixing the LOPEX and ANGERS datasets, randomly taking 80% of the mixed dataset as the training dataset, whereas taking the remaining 20% of the mixed dataset as the validation dataset.*

Their performances were evaluated using the root-mean-square error (RMSE) and coefficient of determination ($R^{2}$). The ML techniques (KNN, PLSR, SVR, and RF) were implemented using the Scikit-Learn package in Python 3.6.

## 3. Results

### 3.1. VI-Based Method (M1) for the Estimation of EWT and LMA

The VI presented in Table 2 were employed to build the statistical relationship between VI and EWT or LMA using a linear or an exponential function. The regressed model with the highest accuracy was selected as the optimal model. Table 3 shows these optimal models under three sampling strategies, i.e., S1, S2, and S3, respectively. The results indicate that the selection of the training dataset is highly important for the VI-based model. Different sampling strategies can result in quite a different optimal regressed model. For example, the MSI-based method provides three exponential models for the estimation of EWT when the S1, S2, and S3 sampling strategies were adopted. As one can see from Table 3, the parameters in these models vary significantly.

**Figure 2:**
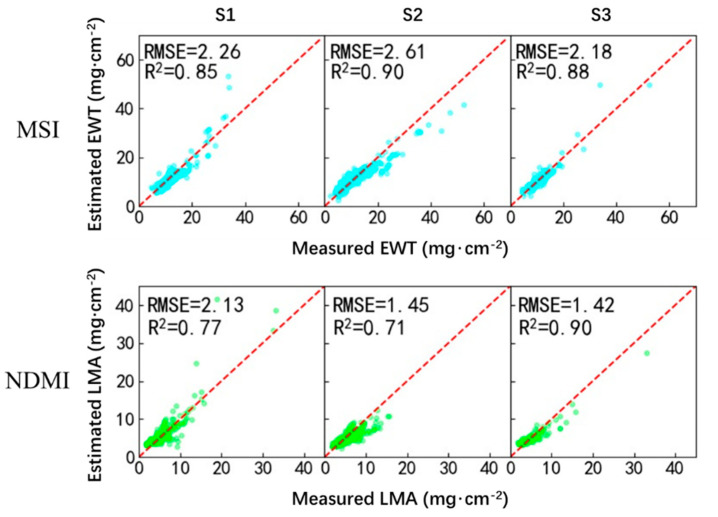
Validation of the moisture stress index (MSI)-based and normalized dry matter index (NDMI)-based models for the estimation of EWT and LMA.

The optimal VI-based models were further validated using the validation dataset, which was selected based on a different sampling strategy (i.e., S1, S2, and S3). Table 4 shows the performances of the validation of the VI-based models. Generally, these models provided satisfactory performance as the estimated and measured EWT and LMA were highly correlated and the corresponding RMSE was relatively small. Among these VI, MSI, and NDMI were the most sensitive indices to EWT and LMA, respectively. The VI-based models adjusted using MSI or NDMI generally provided the best performance in terms of RMSE and $R^{2}$ (i.e., a lower RMSE and a higher $R^{2}$). Figure 2 illustrates the correlation between the estimated and measured EWT and LMA using the two VI-based models (i.e., using MSI for EWT estimation and NDMI for LMA estimation).

### 3.2. ML-Reflectance-Based Method (M2) for the Estimation of EWT and LMA

**Figure 3:**
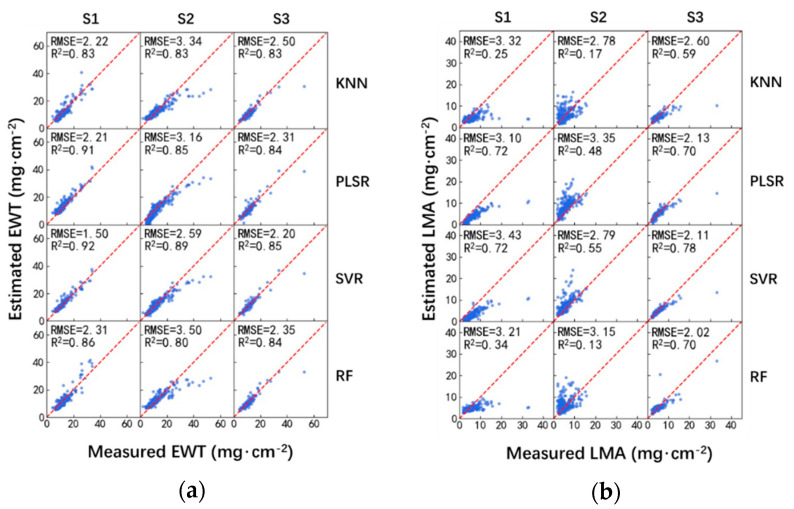
Performance of EWT (**a**) and LMA (**b**) estimation using ML-reflectance-based method.

The machine learning techniques presented in Section 2.3 were utilized for the estimation of EWT and LMA. All the leaf reflectances in the 900–2400 nm spectral region were selected as the inputs to the ML techniques, i.e., each leaf sample has 1501 variables. Figure 3 and Table 5 show the performances of EWT and LMA estimation using the ML-reflectance-based model.

Among these ML techniques, SVR provided the most accurate estimation of EWT under every sampling strategy. Notably, it could significantly outperform the rest of ML techniques when the training and validation datasets were independent, i.e., S1 and S2 sampling strategies. For the S3 sampling strategy, despite that the SVR provided the best performance, the differences of the RMSEs calculated using these ML techniques were not significant, as shown in Figure 3 and Table 5. As for the estimation of LMA, these four ML techniques provided overall comparable performances.

Moreover, the R2 values calculated using the ML-reflectance-based method for EWT estimation were higher than that for LMA estimation. As for the EWT estimation, the R2 were all higher than or equal to 0.8. However, as for the LMA estimation, the R2 values were rarely higher than 0.8, with the highest being around 0.7 or less.

### 3.3. ML-VI-Based Method (M3) for the Estimation of EWT and LMA

**Figure 4:**
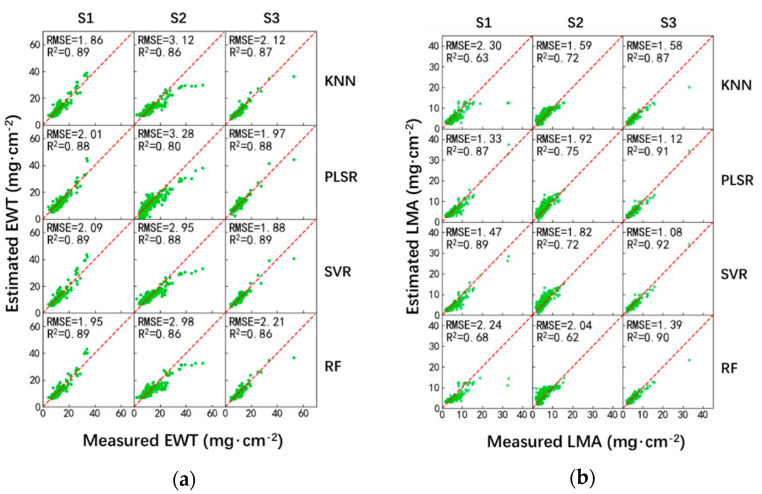
Performance of EWT (**a**) and LMA (**b**) estimation using machine learning (ML)-VI-based method.

The VI presented in Table 2 were employed as inputs of the ML techniques, i.e., the six vegetation indices sensitive to EWT (WI, NDMI, SRWI, NDII, MSI, and DWI) were used for EWT estimation, whereas the four vegetation indices (NDLMA, NDMI, ND, and RI1368,1722) sensitive to LMA were used for LMA estimation. Figure 4 and Table 6 show the performances of EWT and LMA estimation using the ML-VI-based model.

Generally, the performance presented in Table 6 is similar to that in Table 5. Besides the S1 sampling strategy, SVR provided the most accurate estimation of EWT under S2 and S3 sampling strategies. As for the estimation of LMA, PLSR, KNN, and SVR provided the best performances under S1, S2, and S3 sampling strategies, respectively. RF worked less well under these three sampling strategies.

Moreover, the R2 values calculated using the ML-VI-based model for EWT estimation were slightly better than that for LMA estimation. Compared to the ML-reflectance-based model, the R2 values calculated using the ML-VI-based model was improved, especially for the estimation of LMA, as shown in Figure 4 and Table 6.

## 4. Discussion

### 4.1. Performances of the Data-Driven Methods

The performances of the three data-driven methods (M1, M2, and M3) on the remote sensing of EWT and LMA at the leaf level were assessed under three sampling strategies (S1, S2, and S3) in this study. The widely used LOPEX and ANGERS datasets were adopted for the evaluation. Overall, the regressed models using MSI and NDMI provided the best performances in M1 for the estimation of leaf EWT and LMA, respectively (Table 4 and Figure 2); SVR is most effective for the estimation of leaf EWT in M2 and M3; as for the estimation of leaf LMA, the four ML techniques (i.e., KNN, PLSR, SVR, and RF) provides comparable performances in M2 and M3 (Table 5 and Table 6, Figure 3 and Figure 4).

Theoretical and experimental studies have suggested that the influence of leaf biochemical constituents on leaf reflectance and its combination generally does not follow a linear function [18]. According to the optimal VI-based method for the estimation of EWT and LMA in Table 3, an exponential function generally provided the best performance, whereas only in a few cases a linear function outperformed. The exponential functions calibrated using MSI and NDMI were the most recommended functions for the estimation of leaf EWT and LMA, respectively. They provided satisfactory performances under almost every sampling strategy, which were attributed to their strong sensitivity to the corresponding biochemical constituents and insensitivity to other confounding factors [27].

M2 aimed to build a nonlinear relationship between the leaf reflectance in the 900–2400 nm spectral region and the leaf biochemical constituents. Comparison between Table 4 and Table 5 suggested that M2 provided slightly better performance than M1 for the estimation of EWT. Such improvements were attributed to, on one hand, more useful spectral information was added to the training and validation processes in M2, and on the other hand, the ML technique could provide a much more complex nonlinear relationship between the inputs and outputs than an exponential function did [35]. However, for the estimation of LMA, M2 provided much worse performance than M1. Such a result was likely attributed to the predominant water absorption in the 1300–2400 nm spectral region and useless leaf reflectance information in the 900–1300 nm spectral region for LMA estimation [6,17]. It has been reported that the excluding of the 900–1300 nm could improve the performance of LMA estimation [2].

The difference between M2 and M3 was the input to the ML techniques. Table 7 presents the RMSE of M2 and M3 for the estimation of leaf EWT and LMA. Using VI as input to these ML techniques provided improved performances. For EWT estimation, M3 outperformed M2 in 9 cases (12 cases in total). The average RMSE was reduced by 5.7% (=(2.5147 − 2.3703)/2.5147). For LMA estimation, M3 outperformed M2 in all 12 cases. The average RMSE was significantly reduced by 41.5% (=(2.8337 − 1.6581)/2.8337). VI-based methods are mathematical combinations (ratios, differences, and normalized differences, etc.) of reflectances at several bands, with at least one band at which the leaf biochemical material strongly absorbs radiation. The mathematical combination could also suppress the sensitivity of other confounding factors, such as the leaf surface reflection [52]. Therefore, compared to the reflectance at the whole 900–2400 nm spectral region, vegetation indices thus were preferred as the inputs to the ML techniques.

Table 8 presents the comparison of M1 and M3 for the estimation of leaf EWT and LMA. The VI (i.e., MSI and NDMI) and ML technique (i.e., SVR as a representative) that have shown good performances in M1 and M3 were selected. Overall, M3 outperformed M1, i.e., using several VI as inputs to the SVR provided better performance than using a single vegetation index as input to the exponential function for the estimation of both EWT and LMA. The average RMSE indicated that M3 could reduce the error by 1.8% (=(2.3515 − 2.3090)/2.3515) and 12.4% (=(1.6655 − 1.4585)/1.6655) for the estimation of EWT and LMA, respectively. Such results explained that the SVR adopted several more vegetation indices, which could provide additional information about the biochemical constituents, and gave a more complicated nonlinear relationship between the vegetation indices and the biochemical constituents, which was much more accurate than an exponential function. Therefore, M3 is suggested for future estimation of EWT and LMA when using the data-driven methods.

### 4.2. Potential and Limitations of the Data-Driven Methods

Great attention has been devoted to data-driven methods in past decades for the remote sensing of leaf biochemical constituents, especially methods involving ML techniques [17,53]. This study confirmed the good performances of the ML-VI-based method for remote sensing of EWT and LMA. It integrated the advantages of the VI and ML technique, making it insensitive to potential confounding factors and sensitive to the comprehensive nonlinear relationship between VI and leaf biochemical constituents. It thus provided a promising tool for further studies on the remote sensing of leaf biochemical constituents.

Studies have reported that the most sensitive bands may differ with vegetation types and experimental conditions [20,23,45], which consequently impact the value of VI. Therefore, selection of the best wavelength is critical for the ML-VI-based method. Investigations are needed to identify more consistent bands that could be applied to a wide range of vegetation types and experimental conditions. Moreover, with the development of hyperspectral instrumentation and radiative transfer theory, new VI that are sensitive to the change of leaf biochemical constituents may be found and defined [27]. These new VI can introduce more useful information on leaf biochemical constituents, and thus are likely to provide further improvements in the ML-VI-based method.

A mutual drawback of the data-driven method is that its performance is determined by data quality and discrepancies between the training and validation data, which limits its generalization ability when the trained method is applied to different vegetation types or experimental conditions. The discrepancies include, but not limited to, noise level, spectral resolution, and leaf species. It has been reported that the measurements of leaf reflectance in the NIR spectral region might be affected by experimental uncertainties [2,54], such as the noise presented at a higher wavelength in the ANGERS dataset (Figure 1). Different sampling strategies could result in discrepancies between the training and validation dataset [35]. Noise level, presence of outliers and biases, and erroneous data might be different in the training and validation dataset, and thus the three methods (i.e., M1, M2, and M3) gave different performance under three sampling strategies (i.e., S1, S2, and S3), as shown in Table 4, Table 5 and Table 6. Many strategies, such as using expert knowledge for enhancing data quality, and using simulated data during the training stage for reducing data discrepancies, might help to overcome the drawback [2,35]. However, such work has not been well documented and further investigations are needed.

The data-driven methods presented in this study were evaluated using leaf reflectance and its corresponding biochemical constituents at the leaf level. It lays down the foundation for studies that adopting signals collected at the canopy level, such as the Hyperion [55] and AVIRIS [56] hyperspectral spectroradiometers. The applicability of these data-driven methods, especially the ML-VI-based method, at the canopy level needs to be further evaluated. Canopy reflectance models, such as the SAIL [57,58], DART [59,60], and stochastic [61,62] models could be helpful because they bridge the optical properties at different scales, scaling from leaf level up to canopy level. At a higher scale, additional factors, such as the canopy structure, act as confounding factors and should be carefully accounted for. The directional area scattering factor is a canopy structure parameter defined as the canopy BRF if the canopy does not absorb any radiation [63,64]. It can be easily retrieved using measured canopy BRF in the 710–790 nm spectral region without any ancillary information about leaf optics. The directional area scattering factor has been reported to be useful for suppressing the influence of canopy structure on the remote sensing of leaf biochemical constituents [65,66]. It is thus the key to evaluating the data-driven methods on the remote sensing of leaf biochemical constituents at the canopy level.

## 5. Conclusions

In this study, the performances of three types of data-driven methods with different sampling strategies were compared for the estimation of EWT and LMA using leaf reflectances in the 900–2400 nm spectral region. The data-driven methods included the VI-based method (which built a linear or an exponential relationship between VI and leaf biochemical constituents), the ML-reflectance-based method (which built a nonlinear relationship between leaf reflectances and leaf biochemical constituents using ML techniques), and the ML-VI-based method (which built a nonlinear relationship between VI and leaf biochemical constituents using ML techniques). VI that have been reported to be sensitive to leaf EWT and LMA were used, which resulted in the selection of six EWT-related indices (WI, NDWI, SRWI, NDII, MSI, and DWI) and four LMA-related indices (NDLMA, NDMI, ND, RI_1368,1722_). Four ML techniques, i.e., KNN, PLSR, SVR, and RF, were utilized for the representation of the most widely used ML techniques. The independent LOPEX and ANGERS datasets collected over multiple herbaceous woody species were adopted for the evaluation.

Our results showed that the ML-reflectance-based method outperformed the VI-based method for the estimation of EWT. However, it provided a less accurate estimation of LMA than the VI-based method, possibly attributed to the influence of useless leaf reflectance information in the 900–1300 nm spectral region. The ML-VI-based method generally provided better estimations of leaf EWT and LMA than the VI-based method and the ML-reflectance-based method. It inherited the advantage of vegetation indices and ML techniques, which made it sensitive to changes of leaf biochemical constituents and capable of solving nonlinear tasks. Overall, compared to the ML-reflectance-based and VI-based method, the ML-VI-based model with SVR reduced errors by 5.7% (41.5%) and 1.8% (12.4%) for the estimation of leaf EWT (LMA), respectively.

In order to improve the accuracy and generalization ability of the data-driven methods, further investigations are motivated involving the selection of better wavelength, the definition of new vegetation indices, enhancement of the data quality, and reduction of data discrepancies. Moreover, the performances of the ML-VI-based method for the estimation of EWT and LMA at the canopy level needs to be investigated. During such investigations, special attention should be paid because additional confounding factors, such as the canopy structure, may significantly affect the performance of the data-driven methods for remote sensing of leaf biochemical constituents [63,67].

## Figures and Tables

**Table 1:** Statistical characteristics of the experimental datasets.

	LOPEX	ANGERS
Samples	320	276
Species/genotypes	45	43
EWT (mg.cm^−2^)		
Min–max	2.10–52.49	4.39–34.00
Mean ± SD	11.44 ± 6.86	11.62 ± 4.86
LMA (mg.cm^−2^)		
Min–max	1.71–15.73	1.66–33.10
Mean ± SD	5.36 ± 2.48	5.24 ± 3.67

**Table 2:** Vegetation indices used for the estimation of leaf equivalent water thickness (EWT) and leaf mass per area (LMA).

Biochemical Component	Vegetation Index	Formula	Reference
EWT	WI	R_900_/R_970_	[28]
	NDWI	(R_860_ − R_1240_)/(R_860_ + R_1240_)	[29]
	SRWI	R_860_/R_1240_	[30]
	NDII	(R_819_ − R_1600_)/(R_819_ + R_1600_)	[31]
	MSI	R_1600_/R_820_	[32]
	DWI	(R_816_ − R_2218_)/(R_816_ + R_2218_)	[25]
LMA	NDLMA	(R_1368_ − R_1722_)/(R_1368_ + R_1722_)	[23]
	NDMI	(R_1649_ − R_1722_)/(R_1649_ + R_1722_)	[27]
	ND	(R_2295_ − R_1550_)/(R_2295_ + R_1550_)	[23]
	RI_1368,1722_	R_1368_/R_1722_	[22]

**Table 3:** The optimal vegetation indices (VI)-based method for the estimation of EWT and LMA. *x* represents the value of a given vegetation index, and *y* is the corresponding biochemical constituents.

Components	VI	Formula		
		S1	S2	S3
EWT	WI	$y=6e^{23.12x}\times 10^{-10}$	y=492.89x−495.15	$y=3e^{23.53x}\times 10^{-10}$
	NDWI	$y=5.93e^{18.92x}$	$y=4.52e^{23.37x}$	$y=5.55e^{19.44x}$
	SRWI	$y=0.0019e^{8.10x}$	$y=0.0001e^{10.30x}$	$y=0.015e^{8.32x}$
	NDII	$y=3.25e^{5.95x}$	y=84.20x−5.26	$y=3.50e^{5.68x}$
	MSI	$y=307.07e^{-5.03x}$	$y=170.93e^{-4.08x}$	$y=257.87e^{-4.74x}$
	DWI	$y=0.98e^{5.42x}$	$y=1.98e^{3.89x}$	$y=1.39e^{4.66x}$
LMA	ND_LMA_	$y=1.19e^{15.07x}$	y=131.91x−5.29	$y=1.44e^{141.01x}$
	NDMI	$y=1.71e^{47.14x}$	$y=1.84e^{41.37x}$	$y=2.01e^{39.53x}$
	ND	$y=0.58e^{-7.17x}$	y=−41.55x−7.83	$y=0.79e^{-6.11x}$
	RI_1368_	$y=0.0038e^{5.93x}$	y=52.59x−56.57	$y=0.11e^{5.06x}$

**Table 4:** Performance of the VI-based models for the estimation of EWT and LMA. The italic bold number gives the best performances under a given sampling strategy.

Components	VI	S1		S2		S3	
		RMSE	R2	RMSE	R2	RMSE	R2
EWT	WI	3.0244	0.7782	5.5288	0.6821	2.7380	0.8097
	NDWI	3.0205	0.7016	4.4504	0.6376	3.0689	0.7433
	SRWI	3.0393	0.6995	4.6990	0.6116	3.1076	0.7489
	NDII	2.5061	0.8093	2.9805	0.8168	2.5646	0.8523
	MSI	***2.2633***	***0.8535***	***2.6081***	***0.9012***	***2.1831***	***0.8759***
	DWI	3.2537	0.8436	3.2200	0.8553	2.5724	0.8096
LMA	NDLMA	***1.8933***	***0.7915***	2.8150	0.5589	1.8645	0.7801
	NDMI	2.1267	0.7725	***1.4489***	***0.7124***	***1.4209***	***0.8999***
	ND	3.0859	0.3348	2.1180	0.3575	2.9588	0.3843
	RI1368	2.6451	0.7069	2.6956	0.5706	2.1559	0.7454

**Table 5:** Performance of the ML-reflectance-based models for the estimation of EWT and LMA. The italic bold number gives the best performances under a given sampling strategy.

Components	VI	S1		S2		S3	
		RMSE	R2	RMSE	R2	RMSE	R2
EWT	KNN	2.2216	0.8336	3.3370	0.8333	2.4954	0.8257
	PLSR	2.2088	0.9094	3.1585	0.8500	2.3079	0.8395
	SVR	***1.5008***	***0.9208***	***2.5865***	***0.8880***	***2.2022***	***0.8544***
	RF	2.3136	0.8613	3.4974	0.8032	2.3467	0.8353
LMA	KNN	3.3199	0.2531	***2.7787***	0.1682	2.6046	0.5920
	PLSR	***3.0988***	***0.7243***	3.3513	0.4784	2.1280	0.7049
	SVR	3.4337	0.7193	2.7915	***0.5517***	2.1111	***0.7816***
	RF	3.2095	0.3413	3.1539	0.1309	***2.0228***	0.7025

**Table 6:** Performance of the validation of the ML-VI-based models for the estimation of EWT and LMA. The italic bold number gives the best performances under a given sampling strategy.

Components	VI	S1		S2		S3	
		RMSE	*R^2^*	RMSE	*R^2^*	RMSE	*R^2^*
EWT	KNN	***1.8600***	0.8914	3.1195	0.8649	2.1233	0.8669
	PLSR	2.0148	0.8779	3.2849	0.8036	1.9680	0.8839
	SVR	2.0922	0.8905	***2.9502***	***0.8835***	***1.8847***	***0.8940***
	RF	1.9546	***0.8920***	2.9848	0.8601	2.2065	0.8562
LMA	KNN	2.3000	0.6288	***1.5937***	0.7200	1.5829	0.8736
	PLSR	***1.3319***	0.8690	1.9215	***0.7452***	1.1196	0.9087
	SVR	1.4697	***0.8888***	1.8224	0.7239	***1.0833***	***0.9158***
	RF	2.2438	0.6850	2.0376	0.6193	1.3913	0.9033

**Table 7:** RMSE of M2 and M3 for the estimation of leaf EWT and LMA. The italic bold numbers represent better prediction results.

Sampling	ML	M2		M3	
		EWT	LMA	EWT	LMA
S1	KNN	2.2216	3.3199	***1.8600***	***2.3000***
	PLS	2.2088	3.0988	***2.0148***	***1.3319***
	SVR	***1.5008***	3.4337	2.0922	***1.4697***
	RF	2.3136	3.2095	***1.9546***	***2.2438***
S2	KNN	3.3370	2.7787	***3.1195***	***1.5937***
	PLS	***3.1585***	3.3513	3.2849	***1.9215***
	SVR	***2.5865***	2.7915	2.9502	***1.8224***
	RF	3.4974	3.1539	***2.9848***	***2.0376***
S3	KNN	2.4954	2.6046	***2.1233***	***1.5829***
	PLS	2.3079	2.1280	***1.9680***	***1.1196***
	SVR	2.2022	2.1111	***1.8847***	***1.0833***
	RF	2.3467	2.0228	***2.2065***	***1.3913***
Average	-	2.5147	2.8337	***2.3703***	***1.6581***

**Table 8:** RMSE of M1 and M3 for the estimation of leaf EWT and LMA. For M1, RMSEs were calculated using MSI and NDMI-based models, whereas RMSEs were calculated using SVR-VI-based models. The italic bold numbers represent better prediction results.

Sampling	M1		M3	
	EWT (MSI)	LMA (NDMI)	EWT (SVR)	LMA (SVR)
S1	2.2633	2.1267	***2.0922***	***1.4697***
S2	***2.6081***	***1.4489***	2.9502	1.8224
S3	2.1831	1.4209	***1.8847***	***1.0833***
Average	2.3515	1.6655	***2.3090***	***1.4585***

## References

[1] de la Riva E.G., Olmo M., Poorter H., Ubera J.L., Villar R. (2016). Leaf Mass per Area (LMA) and Its Relationship with Leaf Structure and Anatomy in 34 Mediterranean Woody Species along a Water Availability Gradient. PLoS ONE.

[2] Féret J.B., le Maire G., Jay S., Berveiller D., Bendoula R., Hmimina G., Cheraiet A., Oliveira J.C., Ponzoni F.J., Solanki T. (2019). Estimating leaf mass per area and equivalent water thickness based on leaf optical properties: Potential and limitations of physical modeling and machine learning. Remote. Sens. Environ..

[3] Schimel D., Pavlick R., Fisher J.B., Asner G.P., Saatchi S., Townsend P., Miller C., Frankenberg C., Hibbard K., Cox P. (2015). Observing terrestrial ecosystems and the carbon cycle from space. Glob. Chang. Biol..

[4] Jacquemoud S., Ustin S., Verdebout J., Schmuck G., Andreoli G., Hosgood B. (1996). Estimating leaf biochemistry using the PROSPECT leaf optical properties model. Remote. Sens. Environ..

[5] Cheng T., Rivard B., Sánchez-Azofeifa A.G., Féret J.-B., Jacquemoud S., Ustin S.L. (2014). Deriving leaf mass per area (LMA) from foliar reflectance across a variety of plant species using continuous wavelet analysis. ISPRS J. Photogramm. Remote Sens..

[6] Qiu F., Chen J.M., Ju W.M., Wang J., Zhang Q., Fang M.H. (2018). Improving the PROSPECT Model to Consider Anisotropic Scattering of Leaf Internal Materials and Its Use for Retrieving Leaf Biomass in Fresh Leaves. IEEE Trans. Geosci. Remote Sens..

[7] Conejo E., Frangi J.-P., de Rosny G. (2015). Neural network implementation for a reversal procedure for water and dry matter estimation on plant leaves using selected LED wavelengths. Appl. Opt..

[8] Yi Q., Wang F., Bao A., Jiapaer G. (2014). Leaf and canopy water content estimation in cotton using hyperspectral indices and radiative transfer models. Int. J. Appl. Earth Obs. Geoinf..

[9] Baret F., Buis S., Liang S. (2008). Estimating Canopy Characteristics from Remote Sensing Observations: Review of Methods and Associated Problems. Advances in Land Remote Sensing: System, Modeling, Inversion and Application.

[10] Féret J.B., Gitelson A.A., Noble S.D., Jacquemoud S. (2017). PROSPECT-D: Towards modeling leaf optical properties through a complete lifecycle. Remote. Sens. Environ..

[11] Jacquemoud S., Baret F. (1990). PROSPECT: A model of leaf optical properties spectra. Remote. Sens. Environ..

[12] Wang B., Ju W. (2017). Limitations and Improvements of the Leaf Optical Properties Model Leaf Incorporating Biochemistry Exhibiting Reflectance and Transmittance Yields (LIBERTY). Remote Sens..

[13] Dawson T.P., Curran P.J., Plummer S.E. (1998). LIBERTY—Modeling the Effects of Leaf Biochemical Concentration on Reflectance Spectra. Remote. Sens. Environ..

[14] Maier S.W., Lüdeker W., Günther K.P. (1999). SLOP: A Revised Version of the Stochastic Model for Leaf Optical Properties. Remote. Sens. Environ..

[15] Ali A.M., Darvishzadeh R., Skidmore A.K., Duren I.V., Heiden U., Heurich M. (2016). Estimating leaf functional traits by inversion of PROSPECT: Assessing leaf dry matter content and specific leaf area in mixed mountainous forest. Int. J. Appl. Earth Obs. Geoinf..

[16] Li P., Wang Q. (2011). Retrieval of Leaf Biochemical Parameters Using PROSPECT Inversion: A New Approach for Alleviating Ill-Posed Problems. IEEE Trans. Geosci. Remote Sens..

[17] Koirala B., Zahiri Z., Scheunders P. (2020). A Machine Learning Framework for Estimating Leaf Biochemical Parameters from Its Spectral Reflectance and Transmission Measurements. IEEE Trans. Geosci. Remote Sens..

[18] Liu L., Song B., Zhang S., Liu X. (2017). A Novel Principal Component Analysis Method for the Reconstruction of Leaf Reflectance Spectra and Retrieval of Leaf Biochemical Contents. Remote Sens..

[19] Asner G.P., Martin R.E., Tupayachi R., Emerson R., Martinez P., Sinca F., Powell G.V.N., Wright S.J., Lugo A.E. (2011). Taxonomy and remote sensing of leaf mass per area (LMA) in humid tropical forests. Ecol. Appl..

[20] Yao X., Huang Y., Shang G., Zhou C., Cheng T., Tian Y., Cao W., Zhu Y. (2015). Evaluation of Six Algorithms to Monitor Wheat Leaf Nitrogen Concentration. Remote Sens..

[21] Shah S.H., Angel Y., Houborg R., Ali S., McCabe M.F. (2019). A Random Forest Machine Learning Approach for the Retrieval of Leaf Chlorophyll Content in Wheat. Remote Sens..

[22] Féret J.-B., François C., Gitelson A., Asner G.P., Barry K.M., Panigada C., Richardson A.D., Jacquemoud S. (2011). Optimizing spectral indices and chemometric analysis of leaf chemical properties using radiative transfer modeling. Remote. Sens. Environ..

[23] le Maire G., François C., Soudani K., Berveiller D., Pontailler J.-Y., Bréda N., Genet H., Davi H., Dufrêne E. (2008). Calibration and validation of hyperspectral indices for the estimation of broadleaved forest leaf chlorophyll content, leaf mass per area, leaf area index and leaf canopy biomass. Remote. Sens. Environ..

[24] Frampton W.J., Dash J., Watmough G., Milton E.J. (2013). Evaluating the capabilities of Sentinel-2 for quantitative estimation of biophysical variables in vegetation. ISPRS J. Photogramm. Remote Sens..

[25] Colombo R., Meroni M., Marchesi A., Busetto L., Rossini M., Giardino C., Panigada C. (2008). Estimation of leaf and canopy water content in poplar plantations by means of hyperspectral indices and inverse modeling. Remote. Sens. Environ..

[26] Yang B., He Y., Chen W. (2020). A simple method for estimation of leaf dry matter content in fresh leaves using leaf scattering albedo. Glob. Ecol. Conserv..

[27] Wang L.L., Qu J.J., Hao X.J., Hunt E.R. (2011). Estimating dry matter content from spectral reflectance for green leaves of different species. Int. J. Remote Sens..

[28] Penuelas J., Pinol J., Ogaya R., Filella I. (2010). Estimation of plant water concentration by the reflectance Water Index WI (R900/R970). Int. J. Remote Sens..

[29] Gao B.-C. (1996). NDWI—A normalized difference water index for remote sensing of vegetation liquid water from space. Remote. Sens. Environ..

[30] Zarco-Tejada P.J., Rueda C.A., Ustin S.L. (2003). Water content estimation in vegetation with MODIS reflectance data and model inversion methods. Remote Sens. Environ..

[31] Yilmaz M.T., Hunt E.R., Jackson T.J. (2008). Remote sensing of vegetation water content from equivalent water thickness using satellite imagery. Remote Sens. Environ..

[32] Hunt E.R., Rock B.N. (1989). Detection of changes in leaf water content using Near- and Middle-Infrared reflectances. Remote. Sens. Environ..

[33] Wang Z., Wang T., Darvishzadeh R., Skidmore A., Jones S., Suarez L., Woodgate W., Heiden U., Heurich M., Hearne J. (2016). Vegetation Indices for Mapping Canopy Foliar Nitrogen in a Mixed Temperate Forest. Remote Sens..

[34] Axelsson C., Skidmore A., Schlerf M., Fauzi A., Verhoef W. (2013). Hyperspectral analysis of mangrove foliar chemistry using PLSR and support vector regression. Int. J. Remote Sens..

[35] Chlingaryan A., Sukkarieh S., Whelan B. (2018). Machine learning approaches for crop yield prediction and nitrogen status estimation in precision agriculture: A review. Comput. Electron. Agric..

[36] Hosgood B., Jacquemoud S., Andreoli G., Verdebout J., Pedrini G., Schmuck G. (1994). Leaf Optical Properties EXperiment 93 (LOPEX93).

[37] Feret J.-B., François C., Asner G.P., Gitelson A.A., Martin R.E., Bidel L.P.R., Ustin S.L., le Maire G., Jacquemoud S. (2008). PROSPECT-4 and 5: Advances in the leaf optical properties model separating photosynthetic pigments. Remote. Sens. Environ..

[38] Hardisky M.A., Klemas V., Smart R.M. (1983). The influence of soil salinity, growth, form and leaf moisture on the spectral radiance of Spartina alterflora canopies. Photogramm. Eng. Remote Sens..

[39] Gao Y., Lu D., Li G., Wang G., Chen Q., Liu L., Li D. (2018). Comparative Analysis of Modeling Algorithms for Forest Aboveground Biomass Estimation in a Subtropical Region. Remote Sens..

[40] Wei C., Huang J., Mansaray L., Li Z., Liu W., Han J. (2017). Estimation and Mapping of Winter Oilseed Rape LAI from High Spatial Resolution Satellite Data Based on a Hybrid Method. Remote Sens..

[41] McRoberts R.E., Magnussen S., Tomppo E.O., Chirici G. (2011). Parametric, bootstrap, and jackknife variance estimators for the k-Nearest Neighbors technique with illustrations using forest inventory and satellite image data. Remote. Sens. Environ..

[42] Reese H., Nilsson M., Sandstrom P., Olsson H. (2002). Applications using estimates of forest parameters derived from satellite and forest inventory data. Comput. Electron. Agric..

[43] Tomppo E. (2004). Using coarse scale forest variables as ancillary information and weighting of variables in k-NN estimation: A genetic algorithm approach. Remote Sens. Environ..

[44] Jin J., Wang Q. (2019). Evaluation of Informative Bands Used in Different PLS Regressions for Estimating Leaf Biochemical Contents from Hyperspectral Reflectance. Remote Sens..

[45] Hansen P.M., Schjoerring J.K. (2003). Reflectance measurement of canopy biomass and nitrogen status in wheat crops using normalized difference vegetation indices and partial least squares regression. Remote. Sens. Environ..

[46] Asner G.P., Martin R.E., Ford A.J., Metcalfe D.J., Liddell M.J. (2009). Leaf chemical and spectral diversity in Australian tropical forests. Ecol. Appl..

[47] Brown M.P.S., Grundy W.N., Lin D., Cristianini N., Sugnet C.W., Furey T.S., Ares M., Haussler D. (2000). Knowledge-based analysis of microarray gene expression data by using support vector machines. Proc. Natl. Acad. Sci. USA.

[48] Cherkassky V., Ma Y. (2004). Practical selection of SVM parameters and noise estimation for SVM regression. Neural Netw..

[49] Li M., Im J., Quackenbush L.J., Liu T. (2014). Forest Biomass and Carbon Stock Quantification Using Airborne LiDAR Data: A Case Study Over Huntington Wildlife Forest in the Adirondack Park. IEEE J. Sel. Top. Appl. Earth Observ..

[50] Gara T.W., Darvishzadeh R., Skidmore A.K., Wang T., Heurich M. (2019). Accurate modelling of canopy traits from seasonal Sentinel-2 imagery based on the vertical distribution of leaf traits. ISPRS J. Photogramm. Remote Sens..

[51] He Y., Yang B., Lin H., Zhang J. (2020). Modeling Polarized Reflectance of Natural Land Surfaces Using Generalized Regression Neural Networks. Remote Sens..

[52] Li Y., Huang J. (2019). Remote Sensing of Pigment Content at a Leaf Scale: Comparison among Some Specular Removal and Specular Resistance Methods. Remote Sens..

[53] Mountrakis G., Im J., Ogole C. (2011). Support vector machines in remote sensing: A review. ISPRS J. Photogramm. Remote Sens..

[54] Merzlyak M.N., Chivkunova O.B., Melø T.B., Naqvi K.R. (2002). Does a leaf absorb radiation in the near infrared (780–900 nm) region? A new approach to quantifying optical reflection, absorption and transmission of leaves. Photosynth. Res..

[55] Pearlman J.S., Barry P.S., Segal C.C., Shepanski J., Beiso D., Carman S.L. (2003). Hyperion, a space-based imaging spectrometer. IEEE Trans. Geosci. Remote Sens..

[56] Green R.O., Eastwood M.L., Sarture C.M., Chrien T.G., Aronsson M., Chippendale B.J., Faust J.A., Pavri B.E., Chovit C.J., Solis M. (1998). Imaging Spectroscopy and the Airborne Visible/Infrared Imaging Spectrometer (AVIRIS). Remote. Sens. Environ..

[57] Jacquemoud S., Verhoef W., Baret F., Bacour C., Zarco-Tejada P.J., Asner G.P., Francois C., Ustin S.L. (2009). PROSPECT plus SAIL models: A review of use for vegetation characterization. Remote. Sens. Environ..

[58] Verhoef W. (1984). Light scattering by leaf layers with application to canopy reflectance modeling: The SAIL model. Remote Sens. Environ..

[59] Gastellu-Etchegorry J.P., Martin E., Gascon F. (2004). DART: A 3D model for simulating satellite images and studying surface radiation budget. Int. J. Remote Sens..

[60] Gastellu-Etchegorry J.-P., Yin T., Lauret N., Cajgfinger T., Gregoire T., Grau E., Feret J.-B., Lopes M., Guilleux J., Dedieu G. (2015). Discrete Anisotropic Radiative Transfer (DART 5) for Modeling Airborne and Satellite Spectroradiometer and LIDAR Acquisitions of Natural and Urban Landscapes. Remote Sens..

[61] Huang D., Knyazikhin Y., Wang W., Deering D.W., Stenberg P., Shabanov N., Tan B., Myneni R.B. (2008). Stochastic transport theory for investigating the three-dimensional canopy structure from space measurements. Remote. Sens. Environ..

[62] Yang B., Knyazikhin Y., Xie D., Zhao H., Zhang J., Wu Y. (2018). Influence of Leaf Specular Reflection on Canopy Radiative Regime Using an Improved Version of the Stochastic Radiative Transfer Model. Remote Sens..

[63] Knyazikhin Y., Schull M.A., Stenberg P., Mottus M., Rautiainen M., Yang Y., Marshak A., Carmona P.L., Kaufmann R.K., Lewis P. (2013). Hyperspectral remote sensing of foliar nitrogen content. Proc. Natl. Acad. Sci. USA.

[64] Stenberg P., Mõttus M., Rautiainen M. (2016). Photon recollision probability in modelling the radiation regime of canopies—A review. Remote Sens. Environ..

[65] Wang Z., Skidmore A.K., Wang T., Darvishzadeh R., Heiden U., Heurich M., Latifi H., Hearne J. (2017). Canopy foliar nitrogen retrieved from airborne hyperspectral imagery by correcting for canopy structure effects. Int. J. Appl. Earth Obs. Geoinf..

[66] Yang B., Knyazikhin Y., Lin Y., Yan K., Chen C., Park T., Choi S., Mõttus M., Rautiainen M., Myneni R. (2016). Analyses of Impact of Needle Surface Properties on Estimation of Needle Absorption Spectrum: Case Study with Coniferous Needle and Shoot Samples. Remote Sens..

[67] Ustin S.L. (2013). Remote sensing of canopy chemistry. Proc. Natl. Acad. Sci. USA.

